# Hair Metabolomic Profiling of Diseased Forest Musk Deer (*Moschus berezovskii*) Using Ultra-High-Performance Liquid Chromatography–Tandem Mass Spectrometry (UHPLC-MS/MS)

**DOI:** 10.3390/ani15142155

**Published:** 2025-07-21

**Authors:** Lina Yi, Han Jiang, Yajun Li, Zongtao Xu, Haolin Zhang, Defu Hu

**Affiliations:** 1 School of Ecology and Nature Conservation, Beijing Forestry University, Beijing 100083, China; yilnchen@163.com (L.Y.);; 2 Feng County Xinfenghe Animal Hospital Co., Ltd., Baoji 721799, China; 3 Beijing Tong Ren Tang Shaanxi Musk Industry Co., Ltd., Baoji 721004, China; 4 School of Biological Sciences and Technology, Beijing Forestry University, Beijing 100083, China; haolinzhang@bjfu.edu.cn

**Keywords:** *Moschus berezovskii*, forest musk deer, hair, metabolomics, UHPLC-MS/MS, disease

## Abstract

Hair, as a non-invasive biospecimen, retains metabolic deposits from sebaceous glands and capillaries, reflecting substances from the peripheral circulation, and provides valuable biochemical information linked to phenotypes, yet its application in animal disease research remains limited. This work applied ultra-high-performance liquid chromatography–tandem mass spectrometry (UHPLC-MS/MS) to compare the hair metabolomic characteristics of healthy forest musk deer (FMD, *Moschus berezovskii*) and those diagnosed with hemorrhagic pneumonia (HP), phytobezoar disease (PD), and abscess disease (AD). A total of 2119 metabolites were identified in the FMD hair samples, comprising 1084 metabolites in positive ion mode and 1035 metabolites in negative ion mode. Differential compounds analysis was conducted utilizing the orthogonal partial least squares–discriminant analysis (OPLS-DA) model. In comparison to the healthy control group, the HP group displayed 85 upregulated and 92 downregulated metabolites, the PD group presented 124 upregulated and 106 downregulated metabolites, and the AD group exhibited 63 upregulated and 62 downregulated metabolites. Functional annotation using the Kyoto Encyclopedia of Genes and Genomes (KEGG) indicated that the differential metabolites exhibited significant enrichment in pathways associated with cancer, parasitism, energy metabolism, and stress. Receiver operating characteristic (ROC) analysis revealed that both the individual and combined panels of differential metabolites exhibited area under the curve (AUC) values exceeding 0.7, demonstrating good sample discrimination capability. This research indicates that hair metabolomics can yield diverse biochemical insights and facilitate the development of non-invasive early diagnostic techniques for diseases in captive FMD.

## Simple Summary

The population of captive forest musk deer (FMD, *Moschus berezovskii*) is threatened by various diseases, including hemorrhagic pneumonia, phytobezoar disease, and abscess disease, which significantly impact their health and growth. Due to the status of FMD as a protected endangered species, invasive sampling and live animal experiments are conducted under strict legal and regulatory supervision. It is crucial to shift some attention toward research utilizing non-invasive samples. We conducted a comparison of untargeted hair metabolomics data between healthy and diseased FMDs, identifying significantly altered metabolites and functional pathways in the diseased group. The observed differences were found to correlate with the corresponding diseases in the existing literature, and the candidate biomarkers demonstrated capacities for sample classification. In conclusion, non-invasive hair metabolomics presents significant potential for the early detection of FMD diseases and the development of diagnostic tools.

## 1. Introduction

The forest musk deer (FMD, *Moschus berezovskii*) is a small, forest-dwelling ungulate endemic to East Asia. The musk produced by males is a crucial raw material for traditional Asian medicine and global fragrance manufacturing [1]. Due to illicit poaching and habitat fragmentation, the wild FMD population has drastically decreased since the 1950s. In order to restore the FMD population, the Chinese government designated the FMD as a Class I national protected species in 2002 [2]. The IUCN Red List categorized the FMD as “endangered” in 2008 [3]. China initiated the artificial breeding of FMD in 1958 and is the only country globally to have successfully domesticated this species. The FMD is a species characterized by high sensitivity [4]. Factors such as the type of enclosure, farming methods, and feeding operations, etc., can trigger high-stress conditions [5,6,7]. This may lead to a decline in the immune system of FMD, resulting in an increased incidence of diseases such as hemorrhagic pneumonia (HP), phytobezoar disease (PD), and abscess disease (AD) [8,9,10], which pose significant challenges to the growth of FMD breeding populations.

HP is an acute infectious disease that frequently causes mass mortality in FMDs, with infected individuals typically experiencing sudden death without obvious symptoms while exhibiting blood-tinged secretions from the nasal and oral cavities [11]. Pathogens isolated from pulmonary lesions of FMD include *Pseudomonas aeruginosa* [8], *Klebsiella pneumoniae* [12], and *Escherichia coli* [13]. PD originates from indigestion due to densely aggregated plant-fiber phytobezoars, leading to anorexia and emaciation in FMD, ultimately resulting in nutritional exhaustion and fatality due to gastrointestinal obstruction by phytobezoars [10]. AD—the most prevalent disease in FMD—exhibits up to 50% mortality, presenting suppurative lesions on the head, limbs, oral cavity, or digestive tract [14]. The causative agents isolated from AD lesions include *Trueperella pyogenes* [14] and *Bacillus cereus* [15]. Primary pathogens such as *P. aeruginosa* and *T. pyogenes* have established virulence factor profiles and antibiotic susceptibility data [14,16,17,18]. However, development of prevention options for these diseases in captive FMD is hindered by restrictive regulations on invasive experiments due to CITES and Chinese protective laws. Moreover, the shy and evasive nature of FMD hampers the detection of diseases at an early stage. The intense stress response of FMD complicates the operation of health checkups, causing delays in disease diagnosis as well [19]. Hence, there is an urgent need to establish a non-invasive methodology to monitor the long-term biochemical abnormalities associated with disease in FMD.

The temporal scales of the physiological alterations detectable in biological specimens exhibit considerable variability. Blood or urine demonstrates changes occurring within hours, feces indicates alterations over days, and hair offers a record of metabolic products deposited over several months from sebaceous glands and capillaries in hair follicles [20]. In addition to the prolonged biomonitoring window, mammalian hair samples offer significant benefits regarding accessibility and effectiveness. Hair can be obtained through routine care activities, such as plucking during vaccination or collecting naturally shed hair [21]. This practice mitigates the stress responses in nervous animal species [22], lowers the infection risks, and enables long-term, repetitive sampling. Hair is resilient to degradation due to its keratinized structure, improving its transit, storage, and processing efficiency [23]. Therefore, mammalian hair has gained increasing recognition as a practical analytical specimen for current isotope, proteomic, and metabolomic research [24,25,26,27,28].

The metabolome, formed by interactions among the genome, transcriptome, enzyme proteome, and environmental factors, yields sensitive, low-molecular-weight biomarkers (<1500 Da) most relevant to the phenotype [29]. Therefore, the metabolomic characterization of biospecimens has emerged as an important method for investigating the pathophysiological changes induced by diseases [30,31]. Previous metabolomic research has identified novel disease biomarkers in human and animal hair samples, demonstrating significant diagnostic potential. For instance, in the hair of pregnant women with intrahepatic cholestasis, a remarkable impairment of the glutathione metabolic pathway was noted [32]. In rats genetically engineered for Alzheimer’s disease with amyloid-β-induced cognitive deficits, the biosynthesis of phenylalanine and tryptophan was found to be significantly upregulated in their hair [33]. Despite the growing interest in hair metabolomics, this topic remains underexplored within the context of animal disease research.

In light of this, we employed ultra-high-performance liquid chromatography–tandem mass spectrometry (UHPLC-MS/MS) to explore the hair metabolomic features of diseased FMD in cases of hemorrhagic pneumonia (HP), phytobezoar disease (PD), and abscess disease (AD). We further identified disease biomarkers through a comparative analysis between diseased FMD and healthy controls. This study offers a scientific foundation and technical methodology for non-invasive disease diagnosis via hair metabolomics in captive FMD.

## 2. Materials and Methods

### 2.1. Hair Collection

**Figure 1:**
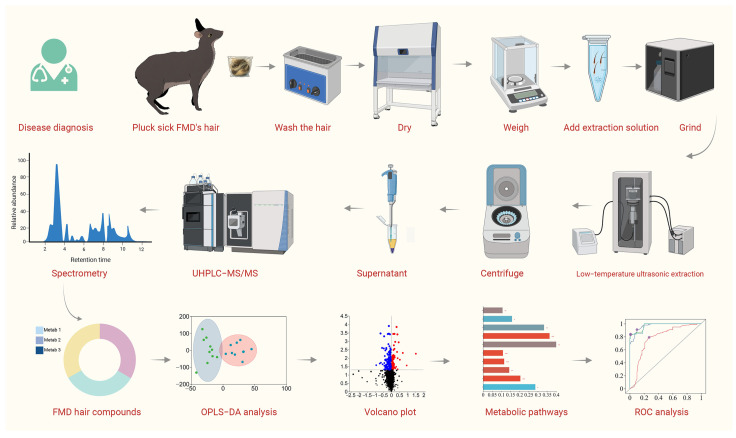
Experimental framework for the untargeted hair metabolomics of captive forest musk deer (FMD) in the present study. The hair samples (hemorrhagic pneumonia, (*n* = 6); phytobezoar disease, (*n* = 6); abscess disease, (*n* = 6); healthy controls, (*n* = 6)) were plucked, washed, dried, weighed, and milled, followed by being subjected to ultrasonic extraction at low temperature using methanol, and subsequently underwent UHPLC-MS/MS analysis. The raw mass spectrometry data were normalized via Progenesis QI software (v3.0). The markedly modified hair metabolites were screened out by a metabolomic workflow to identify potential biomarkers of FMD diseases.

Figure 1 illustrates the comprehensive experimental framework followed in the present study. The collection of FMD hair samples was performed from early February to mid-March 2024 in Feng County, Baoji City, Shaanxi Province, China (E 106°18′ to 108°03′, N 33°35′ to 35°06′). This region holds the largest captive FMD population in China. The subject animals were sourced from FMD farms in the towns of Hekou, Pingmu, and Liufengguan within Feng County.

**Figure 2:**
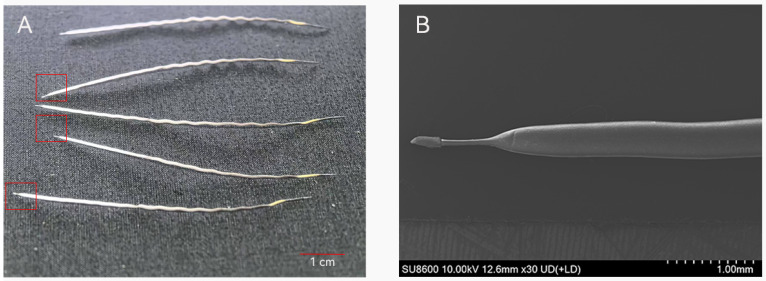
Hair samples from forest musk deer featuring fine, short, spindle-shaped hair roots: (**A**) captured by camera, with red boxes indicating hair follicles; and (**B**) captured by scanning electron microscope.

**Figure 3:**
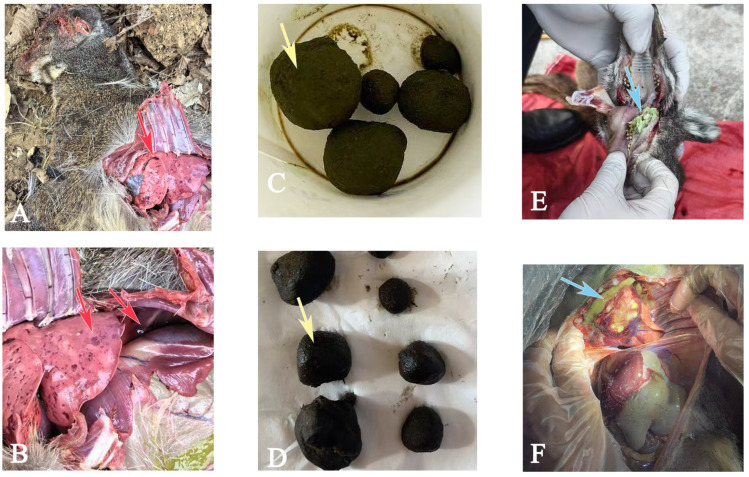
Anatomical examination of lesions in the sampled diseased forest musk deer (FMD). (**A**,**B**) Red arrows indicate pulmonary hemorrhages and pleural effusion in FMD diagnosed with hemorrhagic pneumonia; (**C**,**D**) yellow arrows indicate phytobezoars removed by surgery from the digestive tracts of FMD with phytobezoar disease; and (**E**,**F**) blue arrows indicate oral and visceral abscesses found in FMD with abscess disease.

Hair samples from healthy controls were obtained according to verification from farm personnel that no health issues had been documented in the preceding year. To eliminate disparities between healthy and diseased FMD, samples of healthy controls were obtained on the same day as the diseased FMD samples, from a healthy FMD of comparable age and gender. During the musk collection or vaccination process, when the animals were immobilized, hair samples of healthy individuals were plucked (*n* = 6). As shown in Figure 2, the FMD’s shallow, spindle-shaped hair root makes it prone to shedding. The hair sampling caused no harm or suffering to the FMD. Hair samples of diseased FMD (HP, *n* = 6; PD, *n* = 6; AD, *n* = 6) (Table 1) were obtained from individuals diagnosed by a veterinarian as either surviving or deceased due to the disease (a sample collection interval of ≤ 1 day post-mortem; the impact is negligible because hair growth and metabolism cease after death [34]). Figure 3 illustrates the necropsy findings of the lesions in the sampled diseased FMD. Hair samples from the dorsolateral thigh of FMD were collected (guard hair, ~200 mg, length ≥ 5 cm). Given that an FMD undergoes an annual molt from late May to November, and that poor physiological conditions may impair this process, resulting in partial molting for some individuals [1], we ensured that only recently grown hair, reflecting metabolite deposition over the preceding year, was used for the experiment. We removed the aged hair based on the noticeable color difference—aged hair appears faded and yellowed from extended sun exposure, whereas new hair is darker and shinier—and collected only the newly grown hair for the analysis. The hair samples were stored in sterile ziplock bags and promptly transported to the laboratory for subsequent analysis. The sampled FMD were under the management of Beijing Tong Ren Tang Shaanxi Musk Industry Co., Ltd. (Baoji, China), which approved the sampling procedures in the present study.

### 2.2. Metabolome Extraction

The hair samples underwent washing with isopropanol (analytical grade ≥ 99.5%, Macklin, Shanghai, China) followed by ultrapure water (PURELAB Prima 30, ELGA, High Wycombe, UK), each for 3 min in an ultrasonic bath, to remove surface contaminants [35]. The samples were then placed in a UV-sterilized fume hood for 24 h to ensure complete drying. Approximately 50 mg of dried hair was sectioned into 1 cm snippets using sterilized scissors. Each hair sample was mixed with 400 µL of extracting solvent (methanol: ultrapure water = 4:1, v:v). To monitor the accuracy and reliability of the sample analysis, L-2-chlorophenylalanine (0.02 mg/mL) was used as the internal standard. After grinding for 6 min at −10 °C under a frequency of 50 Hz, ultrasound extraction was performed at 5 °C under 40 kHz for 30 min. The samples were placed in incubators at −20 °C for 30 min, followed by centrifugation of 13,000× *g* at 4 °C for 15 min, after which the supernatants were collected as analytical solutions.

### 2.3. UHPLC-MS/MS Profiling

A Vanquish Horizon UHPLC system (Thermo Fisher, Waltham, MA, USA) equipped with an ACQUITY HSS T3 column (100 mm × 2.1 mm, 1.8 µm, Waters Corporation, Milford, MA, USA) was utilized for the chromatographic separation. The quality control (QC) samples were prepared by blending aliquots of all the FMD hair extractants and following the same experimental procedures as for the test samples. The QC samples were introduced at regular intervals (1 insertion per 5 samples) to assess the stability of the instrument. The column temperature was 40 °C, and the injection volume was 3 μL. The mobile phase consisted of Phase A: acetonitrile and water with 0.1% formic acid (5:95, v:v), and Phase B: acetonitrile, isopropanol, and 5% water with 0.1% formic acid (47.5:47.5:5, v:v:v). The separation gradient (positive ion mode) was as follows: mobile phase B-0%~20% (0~3 min); B-20%~35% (3~4.5 min); B-35%~100% (4.5~5 min); B-100% (5~6.3 min); B-100%~0% (6.3~6.4 min); B-0% (6.4~8 min). The separation gradient (negative ion mode) was as follows: mobile phase B-0%~5% (0~1.5 min); B-5%~10% (1.5~2 min); B-10%~30% (2~4.5 min); B-30%~100% (4.5~5 min); B-100% (5~6.3 min); B-100%~0% (6.4~8 min). The flow rate was 0.4 mL/min. Tandem mass spectrometry was conducted using a Q Exactive HF-X high-resolution mass spectrometer (Thermo Fisher). Electrospray ionization (ESI) was employed to ionize the metabolites eluted from the UHPLC system, functioning at a voltage of ±3500 V in both positive (ESI+) and negative (ESI−) ion modes. The settings for the tandem mass spectrometer were as follows: a base temperature of 425 °C, a sheath gas circulation speed of 50 arb, and an auxiliary gas circulation speed of 13 arb. The data collection relied on data-dependent acquisition (DDA) mode. A mass range of 70~1050 *m*/*z* was scanned at a resolution of 60,000 in the full MS, whereas the MS/MS resolution was established at 7500. The MS/MS mode operated with stepwise collision energy at 20, 40, and 60 au, respectively.

### 2.4. Hair Metabolomic Data Mining

After completing the MS analysis of the sample, the raw metabolic data were imported into the Progenesis QI metabolomic processing software (v3.0, Waters Corporation, Milford, MA, USA), which generated a three-dimensional data matrix in CSV format, containing the sample IDs, metabolite tags, and mass spectral intensities. The internal standard peaks and identified invalid positives (e.g., noise, column bleed, derivatized reagent peaks) were eliminated. Following data filtration, the cleaned metabolic data were cross-referenced against the Majorbio in-house library for standard reference material (Majorbio Bio-Pharm Technology Co., Ltd., Shanghai, China) and the HMDB (http://www.hmdb.ca/, accessed on 11 November 2024), LipidBlast (https://fiehnlab.ucdavis.edu/projects/LipidBlast, accessed on 11 November 2024) and Metlin (http://metlin.scripps.edu, accessed on 11 November 2024) databases. The evaluation criteria for the database search include the mass deviation of primary fragments, isotope similarity, and secondary fragment similarity. The primary mass deviation threshold was set at 10 ppm, and the isotope similarity was set to exceed 30 [36]. For valid results, the fragmentation score (from the in-house standard libraries and spectrometry databases like Metlin and LipidBlast) must exceed 35, or the theoretical fragmentation score (from theoretical databases such as HMDB) must exceed 40 [37,38]. The highest-scoring match is selected as the metabolite identification result. To ensure cross-sample consistency, the intensity of all the detected metabolites was normalized by the total sum value (each value/(sum of each column/coefficient obtained from the largest sum across columns). The coefficient = the total signal intensity of all the peaks in each sample/the maximum sum of signal intensities across all the samples) [36]. Metabolites with a relative standard deviation (RSD) exceeding 30% of the QC samples were excluded. A Log10 transformation was applied to the data matrix. Metabolite features identified in at least 80% of samples within either group were retained. Null values were imputed using the minimum value of the corresponding metabolite.

To detect the hair metabolic alterations in three FMD diseases, principal component analysis (PCA) and orthogonal partial least squares–discriminant analysis (OPLS-DA) were performed using the R package ropls (v 1.6.2). The robustness of the OPLS-DA model was evaluated through 7-fold cross-validation. The volcano plot was created using the R package ggplot2 (v 3.3.0). Significantly differential metabolites were identified based on three criteria, including a variable importance projection score (VIP) > 1, log_2_ fold change > 1, and *p*-value (unpaired Student’s *t*-test) < 0.01. Pathway enrichment analysis of the differential metabolites was conducted using the scipy.stats Python package (v1.0.0). Fisher’s exact test was employed to identify pathways with remarkable enrichment (*p* < 0.05). The enriched pathways were annotated using the Kyoto Encyclopedia of Genes and Genomes Database (KEGG, https://www.kegg.jp/, accessed on 11 November 2024). To evaluate the discriminative capability of the candidate biomarkers, the receiver operator characteristic (ROC) curves were drawn using the R package pROC (version 1.12.1). The sample classification performance of the metabolites was assessed by the area under the curve value (AUC) with a 95% confidence interval (CI).

## 3. Results

### 3.1. Untargeted Metabolomic Characterization of Hair Samples from Captive FMD

**Figure 4:**
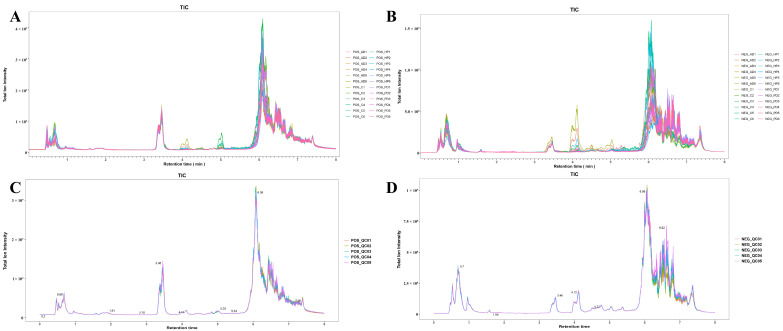
Representative total ion chromatograms (TICs) from collected samples. (**A**,**B**) TICs of experimental samples in positive (**A**) and negative (**B**) ion modes; (**C**,**D**) TICs of quality control samples in positive (**C**) and negative (**D**) ion modes. POS, positive ion mode; NEG, negative ion modes. HP, hemorrhagic pneumonia (*n* = 6); PD, phytobezoar disease (*n* = 6); AD, abscess disease (*n* = 6); C, healthy controls (*n* = 6); and QC, quality control samples (*n* = 5).

After data preprocessing, a total of 5621 valid peaks were detected in positive ion mode (ESI+) and 8090 peaks in negative ion mode (ESI−). The database alignment identified the chemical structures of 1084 and 1035 metabolites for ESI+ mode and ESI− mode, respectively. As depicted in the total ion chromatograms (TICs) in Figure 4A,B, significant differences in the response intensities of various metabolites in the hair samples from the three FMD disease groups (HP1–6, PD1–6, AD1–6) were observed compared to the healthy control samples (C1–C6) in both ESI+ and ESI− modes. The repeatability and stability of the analytical measurements were assessed using quality control (QC) samples. As demonstrated by the TICs of the QC samples in Figure 4C,D, all the QC samples exhibited substantial overlap in terms of the retention time and peak intensity for ion signals. Therefore, the stability and repeatability of the instrumental conditions in this experiment were satisfactory, ensuring that the data quality met the requirements for subsequent analyses.

**Figure 5:**
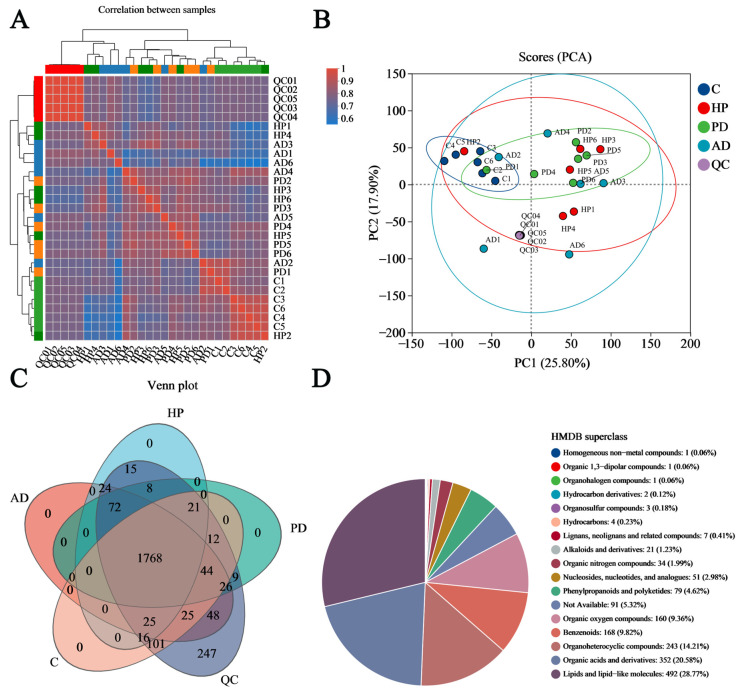
Classification of hair samples from captive forest musk deer based on the metabolomic compositions. (**A**) Correlation heatmap analysis. HP, hemorrhagic pneumonia; PD, phytobezoar disease; AD, abscess disease; C, healthy control; and QC, quality control. (**B**) Principal component plot. (**C**) Venn diagram. (**D**) Metabolite classification derived from the HMDB superclass level.

A correlation heatmap analysis was performed to investigate the relationships across all the samples. As shown in Figure 5A, the strong correlation among the QC samples indicated good experimental reproducibility. Compared to the healthy control samples, the samples from each disease group exhibited reduced correlation and a weaker clustering pattern. We additionally conducted unsupervised principal component analysis (PCA) to examine the sample distribution. As illustrated in Figure 5B, the QC samples were closely gathered in the center of the PCA plot. The samples from the healthy control group displayed clear separation from those in the disease groups. The first and second principal components of the PCA plot accounted for 43.7% of the observed variances, highlighting significant inter-group sample dissimilarity.

As shown in Figure 5C, the Venn diagram indicated that 1768 metabolites were consistently present in all the samples. Compared to the healthy controls, the numbers of unique metabolites identified were 119 for HP, 115 for PD, and 170 for AD. The classification of the compounds in the HMDB database is illustrated in Figure 5D. The percentages of the FMD hair metabolites at different HMDB superclass levels (>1%) in descending order are as follows: lipids and lipid-like molecules (28.77%), organic acids and derivatives (20.58%), organoheterocyclic compounds (14.21%), benzenoids (9.82%), organic oxygen compounds (9.36%), phenylpropanoids and polyketides (4.62%), nucleosides, nucleotides, and analogues (2.98%), organic nitrogen compounds (1.99%), and alkaloids and derivatives (1.23%).

### 3.2. Establishing the OPLS-DA Model

**Figure 6:**
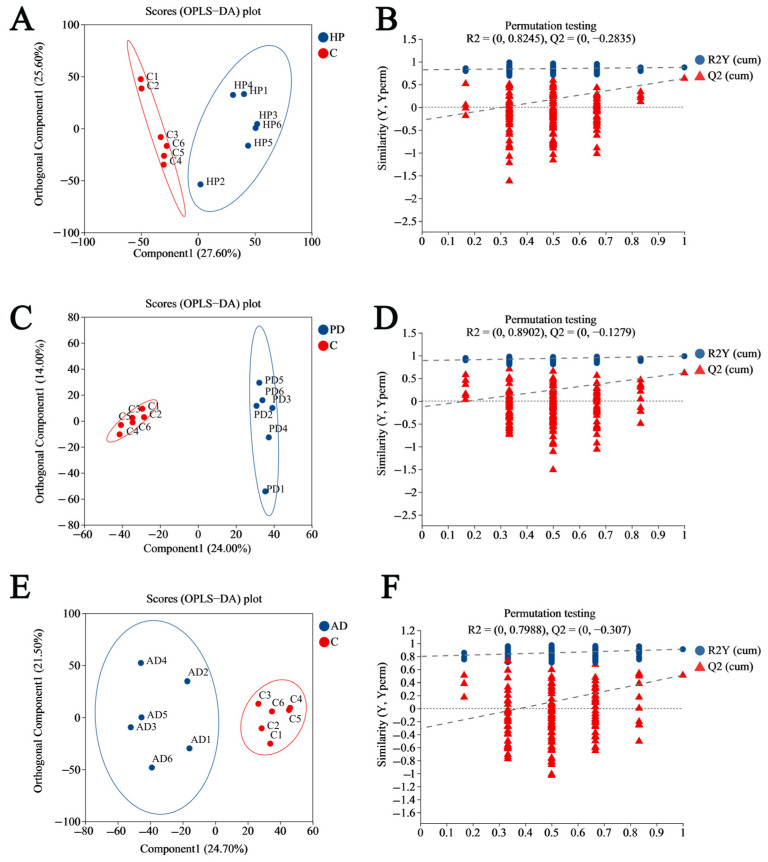
OPLS-DA score plot and permutation test for hair metabolome of healthy and diseased forest musk deer. (**A**,**B**) Hemorrhagic pneumonia vs. healthy control; (**C**,**D**) phytobezoar disease vs. healthy control; (**E**,**F**) abscess disease vs. healthy control. C, healthy controls; HP, hemorrhagic pneumonia; PD, phytobezoar disease; AD, abscess disease.

To further elucidate the inter-group differences and attributable metabolites, we conducted supervised orthogonal partial least squares–discriminant analysis (OPLS-DA) comparing each disease group to the control group. The OPLS-DA score plots exhibited significant sample differentiation in the comparative analysis of HP vs. control (Figure 6A), PD vs. control (Figure 6C), and AD vs. control (Figure 6E) in the mixed ion mode (ESI+ and ESI−). The cumulative R^2^X values for principal components 1 and 2 in the OPLS-DA models of the three diseases were as follows: HP vs. control: R^2^X = 0.53 (>0.5); PD vs. control: R^2^X = 0.38; AD vs. control: R^2^X = 0.46, indicating the principal components of the OPLS-DA model constructed for the HP group most remarkably explained the sample variances. To assess the explanatory capacity of the constructed OPLS-DA models, we calculated the models’ R^2^Y value, Q^2^ value, and Q^2^ intercept after conducting 200 time permutation tests (Figure 6B,D,F). The findings are as follows: HP vs. control: R^2^Y = 0.8245, Q^2^ = 0.635, and Q^2^ intercept = −0.2835; PD vs. control: R^2^Y = 0.8902, Q^2^ = 0.618, and Q^2^ intercept = −0.1279; AD vs. control: R^2^Y = 0.7988, Q^2^ = 0.508, and Q^2^ intercept = −0.307. The results indicate that all the OPLS-DA models exhibit strong discriminative capacity (R^2^Y > 0.5) and predictive capabilities (Q^2^ > 0.5), with no indications of overfitting (Q^2^ intercepts < 0.05).

### 3.3. Screening Differential Hair Metabolites Between Healthy and Diseased Forest Musk Deer

**Figure 7:**
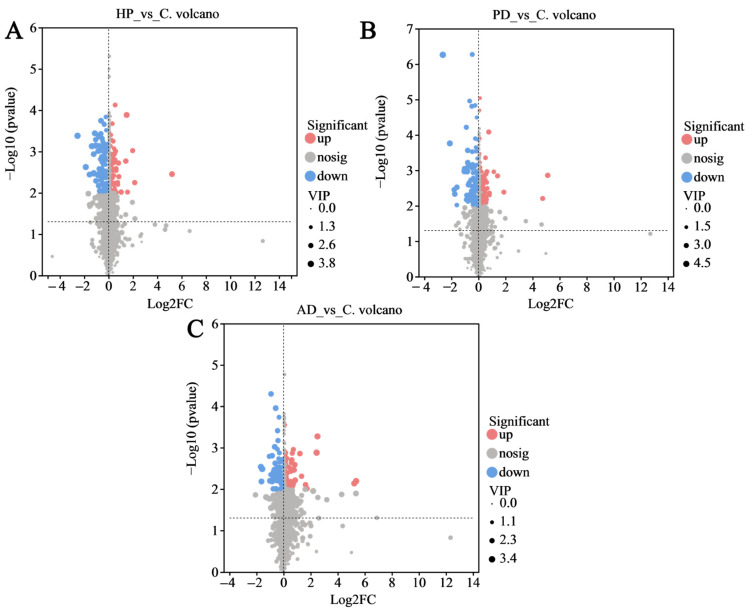
Volcano plots of markedly differential metabolites. (**A**) Hemorrhagic pneumonia vs. healthy control; (**B**) phytobezoar disease vs. healthy control; and (**C**) abscess disease vs. healthy control. Metabolites were selected based on VIP value > 1, log_2_ fold change > 1, and *p* < 0.01. Red bubbles represent upregulation, blue bubbles represent downregulation, and gray bubbles represent no significant difference.

As illustrated in Figure 7, the volcano plot reveals notable changes in the hair metabolomics of diseased FMD compared to healthy individuals (VIP value > 1, log_2_FC > 1, *p* < 0.01). Compared to the healthy control group, the HP group showed 85 metabolites with increased levels and 92 with decreased levels. The PD group had 124 upregulated and 106 downregulated differential metabolites, while the AD group had 63 upregulated and 62 downregulated metabolites.

**Figure 8:**
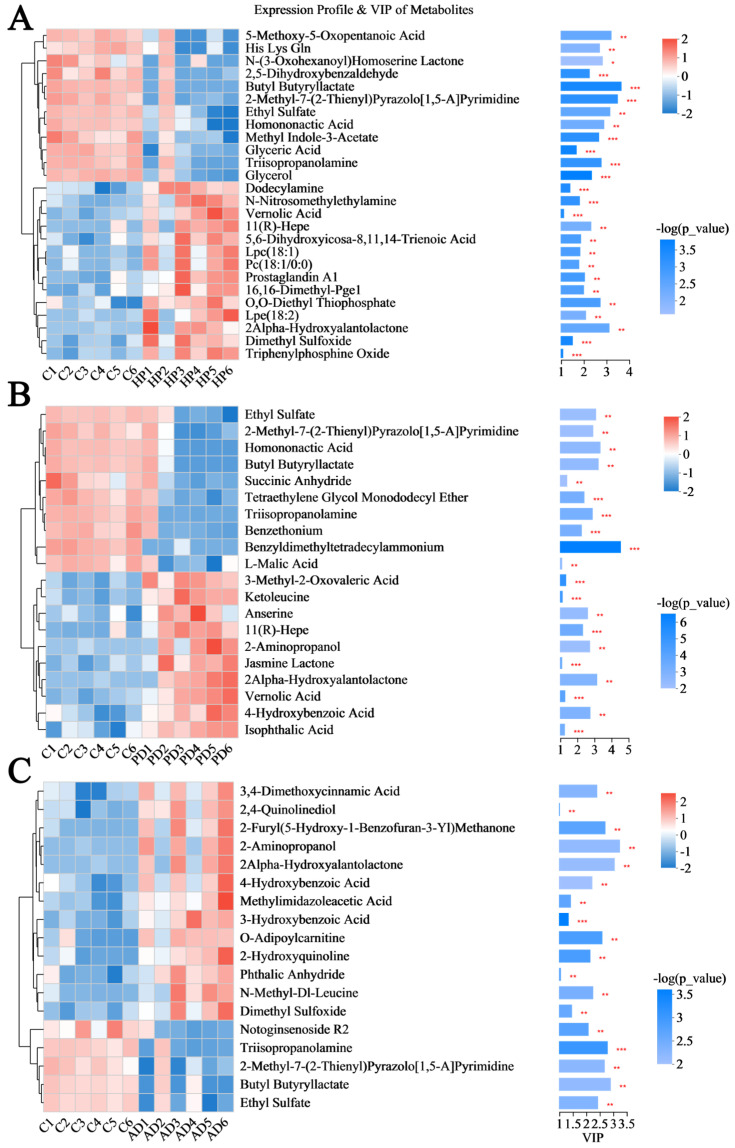
Screening differential hair metabolites between diseased and healthy forest musk deer. (**A**) Hemorrhagic pneumonia vs. healthy controls. (**B**) Phytobezoar disease vs. healthy controls. (**C**) Abscess disease vs. healthy controls. *** *p* < 0.001, ** *p* < 0.01, * *p* < 0.05.

Figure 8 further depicts the metabolites of interest (top10 VIP value, top 10 *p* value, and biologically significant metabolites). As shown in Figure 8A, in comparison to the healthy controls, the relative concentrations of lysophosphatidylcholine (Lpc 18:1) (VIP = 1.83, *p* = 0.002) and lysophosphatidylethanolamine (Lpe 18:2) (VIP = 2.08, *p* = 0.006) were markedly upregulated in the HP samples, while metabolites such as N-(3-Oxohexanoyl) homoserine lactone (VIP = 2.82, *p* = 0.02) exhibited notable downregulation. Figure 8B displays the distinct metabolites identified in the comparison of the PD vs. healthy groups. The PD samples showed significant elevations in metabolites such as 2alpha-hydroxyalantolactone (VIP = 3.14, *p* = 0.001), along with significantly reduced levels of succinic anhydride (VIP = 1.38, *p* = 0.01), L-malic acid (VIP = 1.09, *p* = 0.003), etc. Figure 8C illustrates the differential metabolites detected in the examination of the AD vs. healthy groups, highlighting a remarkable increase in metabolites such as 4-hydroxybenzoic acid (VIP = 2.21, *p* = 0.009) and a notable decrease in triisopropanolamine (VIP = 2.78, *p* = 0.001), etc.

### 3.4. Functional Analysis of Altered Hair Metabolites

**Figure 9:**
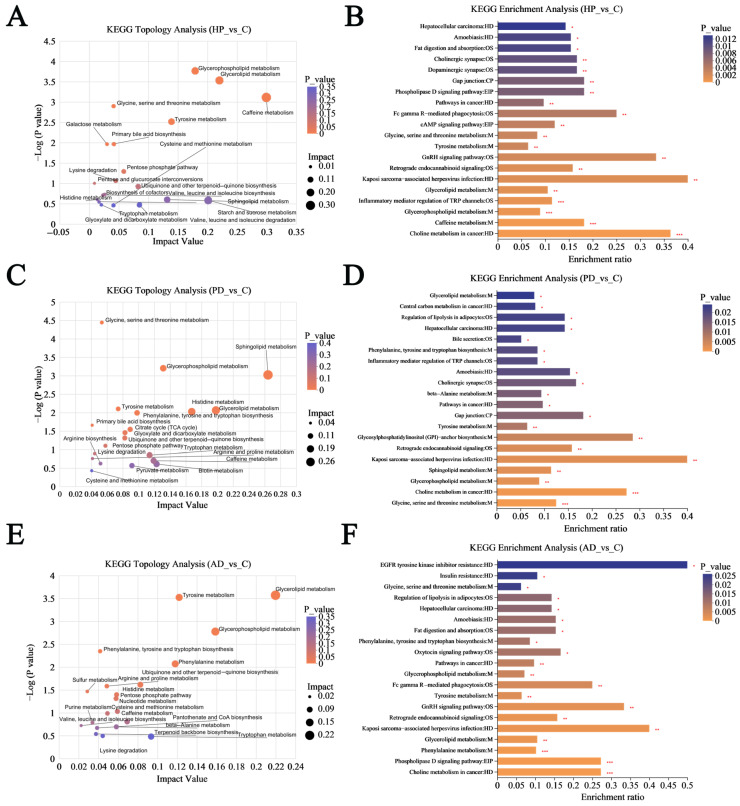
KEGG pathway topological and enrichment analyses of altered metabolites. (**A**,**B**) Hemorrhagic pneumonia vs. healthy control. (**C**,**D**) Phytobezoar disease vs. healthy control. (**E**,**F**) Abscess disease vs. healthy control. KEGG pathway level I abbreviations: OS, Organismal Systems; EIP, Environmental Information Processing; HD, Human Diseases; CP, Cellular Processes; M, Metabolism. *** *p* < 0.001, ** *p* < 0.01, * *p* < 0.05.

To examine the biological functions linked to the altered metabolites, KEGG pathway topological and enrichment analyses were conducted. Figure 9A,B illustrate the results of the analysis comparing the HP samples to the healthy controls. It can be inferred that a total of 35 KEGG level II metabolic pathways were enriched, including “Pathways in cancer” (*p* = 0.006) and “Amoebiasis” (*p* = 0.01). Figure 9C,D present the results from the functional analyses of the PD group. A total of 34 KEGG level II metabolic pathways were identified, which include “Histidine metabolism” (impact value = 0.2), “Lysine degradation” (impact value = 0.04), “Citrate cycle” (impact value = 0.09), and “Pentose phosphate pathway” (impact value = 0.06). Figure 9E,F present the results of the enrichment and topological analysis for the AD samples. A total of 35 KEGG level II metabolic pathways were recognized, including “Retrograde endocannabinoid signaling” (*p* = 0.002) and “Fc gamma R-mediated phagocytosis” (*p* = 0.004).

### 3.5. ROC Analysis of Biomarker Candidates

**Figure 10:**
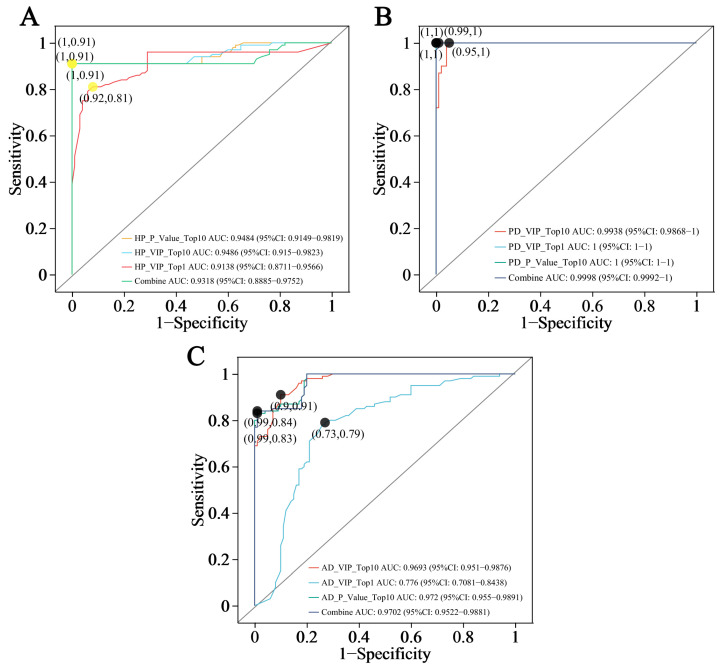
Receiver operating characteristic (ROC) curves for differential hair metabolites between healthy and diseased forest musk deer. (**A**) Hemorrhagic pneumonia vs. healthy control. (**B**) Phytobezoar disease vs. healthy control. (**C**) Abscess disease vs. healthy control. The specificity and sensitivity corresponding to the optimal cutoff point of the ROC curve are labeled on the curves. The area under the curve value (AUC) and 95% confidence interval (CI) are shown beneath all the curves. “Combine” refers to the set of all the tested metabolites.

To assess the classification power of the differential hair metabolites, receiver operating characteristic (ROC) curves were generated. The ROC analysis results of the HP group are illustrated in Figure 10A. The area under the curve (AUC) values for the individual and composite metabolite panels are as follows: top 10 VIP value metabolite set > top 10 *p* value metabolite set > top 1 VIP value metabolite, with an elevated AUC value signifying the biomarker’s enhanced predictive ability for sample discrimination accuracy. The ranking of the AUC’s 95% confidence interval (CI) width is as follows: top 1 VIP value metabolite > top 10 VIP value metabolite set > top 10 *p* value metabolite set. The 95% CI evaluates the model’s stability, with a narrower CI width indicating greater model reliability. The ROC analysis results for the PD group are shown in Figure 10B. The ranking of the AUC values for distinct metabolite sets is as follows: top 10 *p* value metabolite set = top 1 VIP value metabolite > top 10 VIP value metabolite set. The ranking of the 95% CI width of the AUC is as follows: top 10 VIP value metabolite set > top 10 *p* value metabolite set = top 1 VIP value metabolite. Likewise, the ROC analysis of the AD group shown in Figure 10C revealed the following pattern of AUC value ranking: top 10 *p* value metabolite set > top 10 VIP value metabolite set > top 1 VIP value metabolite, and the 95% CI width of the AUC was ordered as follows: top 1 VIP value metabolite > top 10 VIP value metabolite set > top 10 *p* value metabolite set. In summary, while the multivariate ROC curves of the combined metabolite sets improved the AUC values and discrimination compared to the univariate curves of the single metabolites in the comparisons of HP vs. healthy controls and AD vs. healthy controls, this advantage was not observed in the PD group, where both univariate and multivariate panels performed similarly. The ROC analyses showed that all the AUC values for the tested metabolite panels exceeded 0.7, and the lowest lower CI limit was 0.7081, suggesting that both the single and composite metabolite panels based on the VIP or *p* values could distinguish hair samples between healthy and diseased groups, demonstrating diagnostic potential.

## 4. Discussion

This study employed the UHPLC-MS/MS method to conduct a comparative metabolomic analysis of hair samples from healthy and diseased captive FMD. The results revealed that the significantly altered metabolites and functional pathways in the hair of diseased individuals were potentially associated with pathological mechanisms. These findings underscore the research and clinical values of non-invasive hair sampling for long-term biomonitoring in captive-bred protected animal populations.

**Figure 11:**
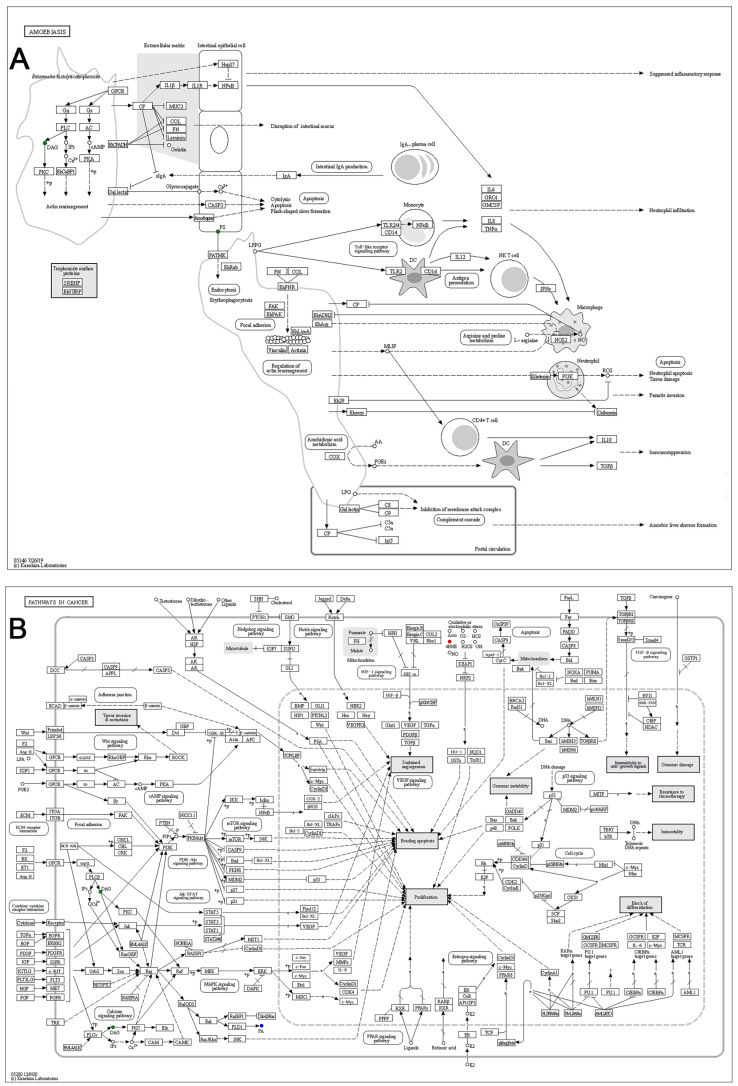

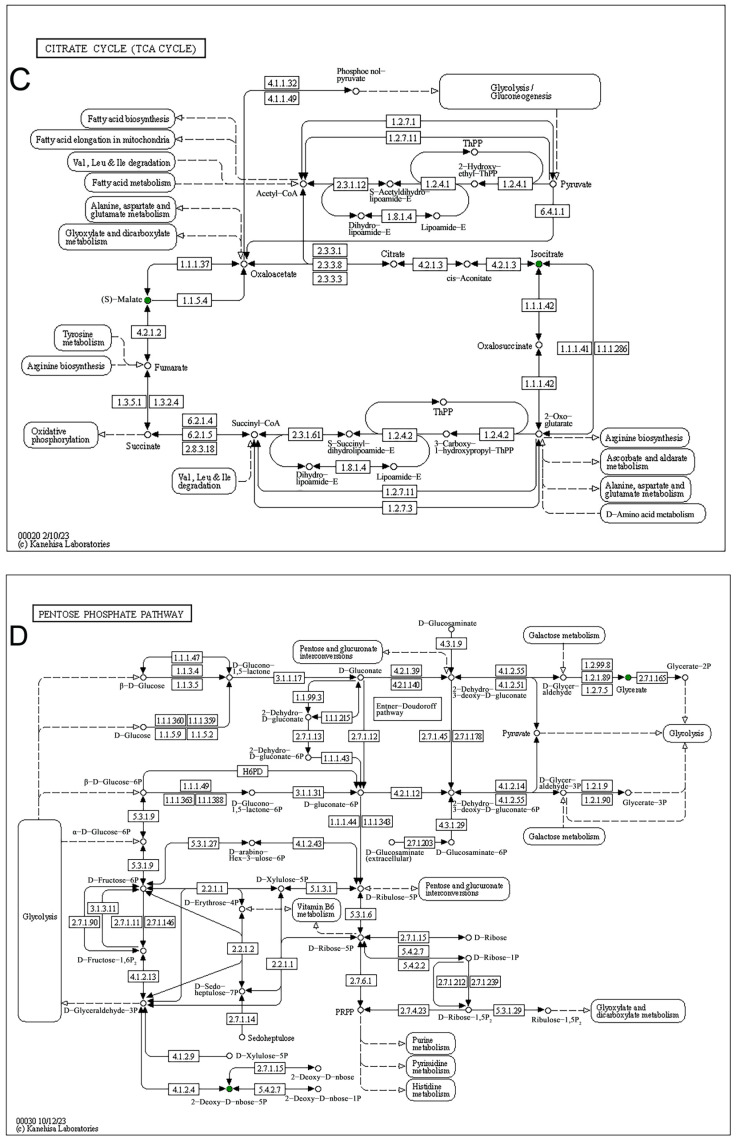

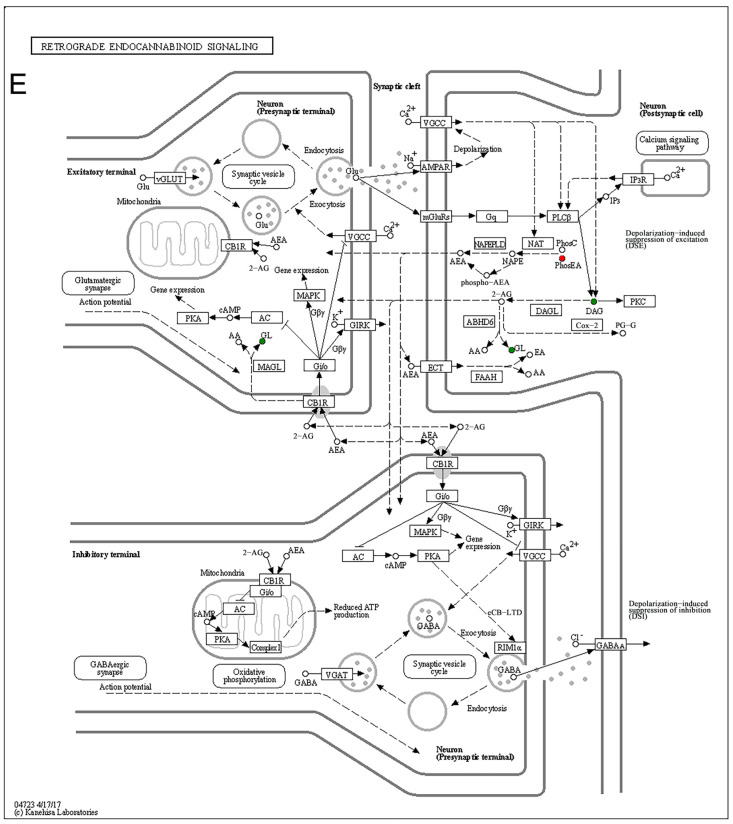
Regulatory networks of metabolic pathways enriched in hair samples from forest musk deer (FMD) with three diseases. For hemorrhagic pneumonia (HP), the (**A**) pathways “Amoebiasis” and (**B**) “Pathways in cancer” are displayed; for phytobezoar disease (PD), the (**C**) “Citrate cycle” (TCA cycle) and the (**D**) “Pentose phosphate pathway” are shown; for abscess disease (AD), the pathway (**E**) “Retrograde endocannabinoid signaling” is presented. Red dots represent considerably increased metabolites in the hair of sick FMD, green dots denote strongly downregulated metabolites, and blue dots show spots where both upregulated and downregulated metabolites coexist.

The metabolomic analysis of the HP hair samples revealed significant alterations in the levels of N-(3-oxohexanoyl) homoserine lactone, a signaling molecule known as N-acyl homoserine lactone (AHL), which has been extensively studied and plays a vital role in the bacterial quorum sensing (QS) system [39,40]. AHL is a self-inducing molecule that triggers QS in Gram-negative bacteria [41]. Previous research showed that the intercellular communication of pathogens through QS systems is essential for the development of infectious diseases [42]. The QS system of *Pseudomonas aeruginosa* enhances its pathogenicity by modulating the expression of virulence factors, such as exoenzymes and toxins that may induce inflammation [43]. Meanwhile, multi-antibiotic-resistant *P. aeruginosa* has frequently been isolated from the lung tissues of FMD individuals affected with HP [8,17,44]. The findings of the current investigation indicated that the AHL levels in the hair of the HP group were markedly lower than those in the healthy control group, underscoring that in vivo modifications to the AHL levels may be intricately associated with the course of HP. The downregulation of AHL in the hair of HP individuals may be caused by downstream effects or feedback regulation. Experimental validation utilizing model animals will help to clarify this phenomenon. Among other metabolites, lysophosphatidylethanolamine LPE (18:2) and lysophosphatidylcholine LPC (18:1) emerge as compounds of particular interest [45,46]. Ma et al. reported that the serum LPC concentrations and LPE-related ratios effectively predict the disease severity and 30-day mortality in community-acquired pneumonia (CAP) patients [45]. Furthermore, Li et al. demonstrated that incorporating LPE acyltransferase (LPEAT) measurements into clinical scoring systems significantly improves the prognostic accuracy [47]. Building on our observed alterations in the LPC and LPE levels in the HP samples, these lysophospholipids may also be closely linked to pneumonia progression in FMD. Additional studies are needed to confirm this association. The functional analysis demonstrated that the pathways of “Amoebiasis” and “Pathways in cancer” were considerably enriched in the HP samples relative to the healthy controls. Furthermore, this discovery aligns with a previous proteomics study on lung tissue from three FMD who died of pneumonia [48], which also demonstrated enrichment in the “Amoebiasis” and “Pathways in cancer” pathways. The authors of the proteomic analysis speculated that analogous or shared immune response pathways may be present between FMD pneumonia and amoebiasis or cancer. The findings from the current hair metabolome analysis of FMD with hemorrhagic pneumonia have validated their proteomic results. The enriched metabolites in these pathways are closely involved in FMD’s immune regulation. For instance, in the “Amoebiasis” pathway, phosphatidylserine (PS) translocation across the plasma membrane serves as an early apoptotic marker, which can be recognized by extracellular receptors to initiate phagocytosis [49] (Figure 11A). The diacylglycerol (DAG) and phosphatidic acid (PA) within this pathway can regulate protein kinase C (PKC) activity, thereby influencing cellular proliferation and survival [50] (Figure 11A). In “Pathways in cancer”, the accumulation of 4-hydroxynonenal (4HNE) reflects intracellular oxidative stress, which may disrupt PKC signaling by altering the DAG/PA levels [51] (Figure 11B). Alterations in these metabolites can constitute pathogenic or immunological mediators of HP, suggesting their potential utility as diagnostic indicators or therapeutic targets.

Metabolic analysis of the PD hair samples demonstrated significant downregulation of features related to energy metabolism, including succinic anhydride (a dehydrated form of succinic acid, and succinic acid acts as a substance in the mitochondrial electron transport chain [52]) and L-malic acid (a vital TCA cycle intermediate [53]). Functional analysis revealed that the pathways within KEGG level I, “Metabolism”, exhibited a substantially higher enrichment in the PD samples (35.4%) than in the AD samples (31.1%) and the HP samples (27.3%). The specific pathways enriched in the PD samples within the “Metabolism” category are closely linked to carbohydrate metabolism, such as the “Citrate cycle” (TCA cycle) and the “Pentose phosphate pathway”. It is widely agreed that the TCA cycle is the fundamental pathway of cellular oxidative phosphorylation, essential for energy metabolism, biosynthesis, and redox homeostasis in cells and organs [54]. In the PD samples, the specific metabolites decreased within the TCA cycle include isocitrate and malate (Figure 11C). The isocitrate drives cycle progression via oxidative decarboxylation to α-ketoglutarate, while malate regenerates oxaloacetate to restart the cycle [55]. The pentose phosphate pathway functions as a derivative of glycolysis in the initial stage of carbohydrate metabolism, is essential for ribonucleotide synthesis, and serves as the main provider of NADPH [56]. In addition, the downregulated metabolites in PD, including glycerate and 2-deoxy-D-ribose-5-phosphate derivatives (G3P/F6P), provide rapid energy substrates by feeding into glycolytic intermediates during periods of heightened energy demands [57,58] (Figure 11D). Previous studies have shown that in the case of intestinal inflammation, a significant increase in energy consumption occurs due to the high energy demand for promoting cell division and repairing damaged mucosa, which leads to lower levels of TCA-cycle-related molecules [59,60]. Therefore, the declines of the metabolites within the energy metabolism pathways in the PD samples may also be a compensatory mechanism in FMD to meet the energy needs under conditions of digestive tract obstruction, which requires further experimental validation.

By examining the differential metabolites set of the AD group, we identified 4-hydroxybenzoic acid as a key compound. 4-hydroxybenzoic acid has been reported to be positively correlated with *Shigella* infection in the gut and is involved in this pathogen’s QS mechanism [61]. Elevated levels of 4-hydroxybenzoic acid were also detected alongside pathogenic bacteria in mice with sepsis [62]. Therefore, the presence of 4-hydroxybenzoic acid in the FMD hair samples from the AD group may be derived from pathogenic metabolic byproducts as well. Furthermore, our functional pathway analysis revealed alterations related to stress levels in the diseased FMD hair samples, as the “Retrograde endocannabinoid signaling” pathway was significantly enriched in the HP, PD and AD hair samples. Previous studies have documented the regulatory role of the endocannabinoid system in animals’ stress response [63]. The activity of cannabinoid receptors could significantly influence the hypothalamic–pituitary–adrenal (HPA) axis and directly regulate cortisol release [64]. In “Retrograde endocannabinoid signaling”, the significantly differential DAG in the AD samples can be converted into 2-arachidonoylglycerol (2-AG) via DAGL α/β, activating presynaptic cannabinoid receptor 1 (CB1) to modulate synaptic plasticity and stress responses [65] (Figure 11E). 2-AG is an important endocannabinoid controlling the release of neurotransmitters. This process plays an important role in synaptic plasticity, neural regulation, and stress response [66]. The spatiotemporal regulation of 2-AG signaling is tightly controlled by MAGL-mediated degradation and ABHD6-driven hydrolysis [67], with MAGL and ABHD6 also being observed with remarkable changes in the AD samples (Figure 11E). The cortisol levels in hair and plasma samples were found to be significantly correlated with the 2-AG and anandamide (AEA) contents [68,69]. This is consistent with previous studies that have extensively recognized altered cortisol levels in the hair and feces of FMD as reliable indicators of significant physiological stress [70,71]. Such stress in captive FMD is largely due to a conflict between their solitary biological nature and the constraints of captivity, which is further exacerbated by their retained wildness from a brief domestication history. In line with this, our hair metabolome analysis supports the existing literature that links excessive stress responses and chronic cortisol release to physiological “exhaustion” and impaired immune function in FMD [6,7,71]. Consequently, this results in increased disease susceptibility, which was notably observed in the sick captive FMD individuals in the present study.

Our study is the first comparative analysis of the untargeted hair metabolomics in healthy and diseased FMD, revealing that hair specimens hold powerful biomarkers. The AUC values in the ROC analysis for all the tested single and composite metabolite sets surpassed 0.7, indicating significant diagnostic potential for differential metabolites exhibiting high VIP or *p* values in the comparative analysis. The FMD is classified as an endangered species. Consequently, biological experiments involving live FMD are strictly prohibited, and invasive sampling necessitates an elaborate animal ethics review. The symptoms of FMD diseases are often difficult to detect and progress rapidly. The collection of samples from FMD that died of disease often exhibits a notable time delay, leading to degradation of the DNA and RNA, thereby obstructing a full investigation of FMD pathophysiology. In contexts where invasive sampling is challenging, non-invasive samples like hair and feces have proven to be valuable biospecimens in FMD studies [6,72]. Furthermore, for FMD diseases characterized by prolonged courses with unnoticeable symptoms, such as phytobezoar disease and abscess disease, hair provides a monitoring window that extends over several months, making it an exceptionally appropriate sample type [73]. The limited sample size (only six samples per group) may compromise the generalizability of our findings and lead to inflated AUC values. Future multi-center, large-sample collaborative research is necessary to validate the disease-associated biomarkers identified in this study. Furthermore, targeted metabolomics analysis should be employed for a deeper exploration of these biomarkers, and their biological functions need to be validated through studies in laboratory animals. RNA extraction from FMD hair follicles for transcriptome sequencing was attempted; however, the results were unsatisfactory due to the insufficient RNA quality and quantity. As a pilot study, this research serves as an initial step. Future research may focus on improving the sample processing protocol to obtain high-quality genetic material from hair follicles, as well as validating the correlation between hair metabolites and plasma/tissue metabolites. Incorporating multi-omics approaches, such as genomics, transcriptomics, and proteomics, could help to comprehensively analyze the non-invasive disease markers in FMD. Relative studies will serve as an effective research model for disease precautions in domesticated wild animals.

## 5. Conclusions

In summary, this study represents the first comparative investigation of the hair metabolome between healthy and diseased captive FMD. We identified significantly altered metabolites in the hair of FMD suffering from hemorrhagic pneumonia, phytobezoar disease, and abscess disease compared to healthy individuals. Some of these metabolites have been previously reported as biomarkers for the corresponding diseases or pathogens. In the ROC analysis, both the individual and composite metabolite panels demonstrated good sample classification performance. Functional analysis revealed that the differential metabolites were significantly enriched in pathways related to cancer, parasitism, energy metabolism, and physiological stress. Future studies should employ larger sample sizes, include experimental animal models, and cross-reference diverse tissue samples to validate the hair biomarkers identified in this study. The findings highlight the potential of non-invasive hair sampling combined with untargeted metabolomics as a promising tool for biomonitoring and early disease diagnosis in captive FMD.

## Figures and Tables

**Table 1:** Individual information and descriptions of the forest musk deer sampled in this study.

ID	Age (Years)	Gender	Description of Conditions
HP1	0.7	♀	Hemorrhagic pneumonia
HP2	3	♂	Hemorrhagic pneumonia
HP3	0.7	♂	Hemorrhagic pneumonia, infant, infected from mother in the same enclosure
HP4	2	♀	Hemorrhagic pneumonia
HP5	3	♂	Hemorrhagic pneumonia
HP6	2	♀	Hemorrhagic pneumonia, pregnant female
PD1	2	♀	Anorexia, rumen indigestion, presence of phytobezoars
PD2	4	♀	Indigestion, chronic fecal impaction with undigested fibers, phytobezoars
PD3	2	♀	Death due to gastrointestinal blockage induced by phytobezoars
PD4	6	♂	Anorexia, vomiting, gastrointestinal obstruction caused by phytobezoars
PD5	2	♂	Gastrointestinal obstruction caused by phytobezoars
PD6	4	♂	Phytobezoar-induced gastrointestinal disease, anorexia, sudden death during treatment
AD1	1.5	♀	Abscess disease, continuous weight loss
AD2	0.7	♂	Post-mortem examination revealed abscesses in the lungs, sudden death in infant
AD3	1.8	♀	Greenish pus in the leg, continuous weight loss
AD4	7	♂	Post-mortem examination revealed abscesses in the thigh muscles and lungs
AD5	4	♂	Visible abscess in the leg
AD6	7	♂	Visible abscess in the leg
C1	0.7	♀	Healthy, no record of past illness
C2	2	♀	Healthy, no record of past illness
C3	4	♂	Healthy, no record of past illness
C4	3	♂	Healthy, no record of past illness
C5	2	♂	Healthy, no record of past illness
C6	6	♂	Healthy, no record of past illness

## Data Availability

The original contributions presented in this study are included in the article. Further inquiries can be directed to the corresponding author.
